# Reduction of the serotonin 5-HT_1B_ and 5-HT_2A_ receptor-mediated contraction of human pulmonary artery by the combined 5-HT_1B_ receptor antagonist and serotonin transporter inhibitor LY393558

**DOI:** 10.1007/s43440-020-00105-2

**Published:** 2020-04-24

**Authors:** Marta Baranowska-Kuczko, Hanna Kozłowska, Eberhard Schlicker, Manfred Göthert, Margaret R. MacLean, Mirosław Kozłowski, Monika Kloza, Olga Sadowska, Barbara Malinowska

**Affiliations:** 1 Department of Experimental Physiology and Pathophysiology, Medical University of Bialystok, Mickiewicz Str. 2A, 15-089 Bialystok, Poland; 2 Department of Clinical Pharmacy, Medical University of Białystok, Białystok, Poland; 3 Department of Pharmacology and Toxicology, University of Bonn, Bonn, Germany; 4 Strathclyde Institute of Pharmacy and Biomedical Sciences, University of Strathclyde, Glasgow, UK; 5 Institute of Cardiovascular and Medical Sciences, University of Glasgow, Glasgow, UK; 6 Department of Thoracic Surgery, Medical University of Białystok, Białystok, Poland

**Keywords:** 5-HT_1B_ receptor, 5-HT_2A_ receptor, LY393558, Pulmonary arterial hypertension, Serotonin, Serotonin transporter

## Abstract

**Background:**

LY393558 is a combined antagonist of serotonin (5-HT) 5-HT_1B_ receptors and inhibitor of serotonin transporter (SERT). LY393558 reduces 5-HT-induced vasoconstriction and remodelling of rat and/or mouse pulmonary arteries. The aim of our study was to examine the effect of LY393558 on the 5-HT-stimulated vasoconstriction of human pulmonary arteries (hPAs) and to determine the underlying mechanism(s).

**Methods:**

Vascular effects of 5-HT receptor agonists, antagonists and a SERT inhibitor were examined in organ bath studies on intralobar hPAs obtained from patients during resection of lung carcinoma.

**Results:**

Serotonin and agonists of the 5-HT_1B_ receptor (5-carboxamidotryptamine, 5-CT) and 5-HT_2A_ receptor (α-methyl-5-HT) contracted endothelium-intact hPAs in a concentration-dependent fashion. The 5-HT_1B_ antagonists SB224289 and GR55562 reduced responses induced by 5-HT and 5-CT and the 5-HT_2A_ antagonist ketanserin inhibited the effects of 5-HT and α-methyl-5-HT. Administration of the SERT inhibitor citalopram (at a concentration that failed to modify the 5-HT-induced vasoconstriction) in combination with SB224289 or GR55562 was more effective in inhibiting the response to 5-HT than the 5-HT_1B_ antagonists alone. LY393558 showed the greatest antagonistic effect against the vasoconstriction elicited by 5-HT, 5-CT and α-methyl-5-HT.

**Conclusions:**

LY393558 reduces the 5-HT-induced contraction antagonizing 5-HT_1B_ and 5-HT_2A_ receptors probably due to synergic interaction between SERT inhibition and 5-HT_1B_ receptor antagonism. Thus, it might represent a valuable future option in the pulmonary arterial hypertension therapy.

## Introduction

Pulmonary arterial hypertension (PAH) is characterized by vascular constriction, proliferation, fibrosis and inflammation. The exact pathophysiology of PAH is unknown and there is a need for novel therapeutic targets [1–3]. The PAH outbreak in people taking anorexigens that are indirect serotonergic agonists and may increase serotonin (5-hydroxytyramine, 5-HT) plasma levels led to the serotonin hypothesis of this disease. Serotonin induces both a vasoconstriction of pulmonary arteries (PAs) mainly via 5-HT_1B_ and 5-HT_2A_ receptors (5-HT_1B_Rs and 5-HT_2A_Rs) and promotes smooth muscle proliferation predominantly dependent via the 5HT_1B_R and the serotonin transporter (SERT) [4–6]. The following facts observed in patients and/or animal models with PAH confirm the involvement of 5-HT in PAH pathophysiology: (1) higher plasma concentration of free 5-HT connected with lower platelet 5-HT storage [7]; (2) higher expression of SERT and tryptophan hydroxylase 1 (TPH1) than in controls [7]; (3) protection or reversal of experimental PAH by genetic ablation and/or pharmacological inhibition of TPH1, SERT or 5-HT_1B_Rs [1, 2, 8]; (4) mice over-expressing the gene for human SERT and SERT knockout mice are more and less susceptible to hypoxia-induced PAH, respectively [9–11]; (5) greater sensitivity of Fawn-Hooded rats (FH, which have a genetic defect in 5-HT platelet storage) than Sprague–Dawley (SD) controls to hypoxia-induced PAH [12].

Targeting SERT plus 5-HT_1B_Rs may become a novel therapeutic approach to PAH [2, 13]. Although SERT inhibitors increase 5-HT levels and subsequently lead to 5-HT receptor activation, synergic activity between SERT and 5-HT_1B_R inhibitors against vasoconstriction and proliferation has been shown in the pulmonary circulation [12, 14]. Thus, the combined inhibitory effects of the SERT inhibitor fluoxetine (at a concentration that has no inhibitory effect of its own) and the 5-HT_1B_R antagonist SB224289 on the 5-HT-induced vasoconstriction of PAs isolated from SD were more than additive [12]. LY393558, a combined 5-HT_1B_R antagonist and SERT inhibitor [15, 16], exerted a more potent effect than SB224289 and fluoxetine given separately. In addition, it inhibited the 5-HT-induced constriction of PAs from FH and hypoxic FH and SD rats and the proliferation of human pulmonary artery smooth muscle cells (hPASMCs) derived from idiopathic PAH patients [12, 13]. LY393558 was more effective in PAH prevention and reversal in two mouse models of pulmonary hypertension than the SERT inhibitor citalopram [13].

The aim of the present study was to examine the effect of LY393558 on the vasoconstriction of human pulmonary arteries induced by 5-HT. To determine the effect of LY393558 in more detail we extended our experiments to 5-HT_1B_R and 5-HT_2A_R ligands and the SERT inhibitor citalopram.

## Materials and methods

Experimental protocols were approved by the Human Ethics Committee of the Medical University of Białystok, Poland. The tissue donors provided written informed consent for the use of their blood vessels.

Lung tissue was received from 29 men and 5 women (mean age 65 ± 2 years) with lung carcinoma (without PAH) undergoing lobectomy or pneumonectomy. Before the operation, they received cephalosporins and heparin as anti-infection and antithrombotic prophylaxis, respectively. Isolation of hPAs has been described previously [17]. When necessary, endothelium was disrupted by rubbing of the lumen with horse hair. Arterial rings (3–5 mm in length and 2–4 mm in outer diameter) were mounted in 10 ml organ baths containing Tyrode’s solution [17] gassed with 95% O_2_ and 5% CO_2_ (37 °C, pH 7.4). Resting tension ranged from 19.6 to 24.5 mN (equivalent to transmural pressure of ~ 16 mmHg). Muscle tension was recorded by a force displacement transducer (BIO-SYS-TECH). After equilibration, rings were exposed to high KCl (60 mM) Tyrode’s solution (equimolar substitution of NaCl by KCl) to check tissue viability and maximum contraction and to phenylephrine (1 µM) followed by acetylcholine (10 µM) to verify if the endothelium is intact. Rings were contracted with 5-HT or the agonists 5-CT (5-HT_1B/1D_R), α-methyl-5-HT (5-HT_2A_R), phenylephrine (α_1_-adrenoceptor) and U46619 [thromboxane A_2_ (TP) receptor] and cumulative concentration–response curves (CRCs) were constructed. The antagonists GR55562 (5-HT_1B/1D_R), SB224289 (5-HT_1B_R), ketanserin (5-HT_2A_R), the SERT inhibitor citalopram and the combined 5-HT_1B_R antagonist and SERT inhibitor LY393558 were incubated for 45 min and also present during construction of CRCs. Concentrations of antagonists and citalopram were taken from previous studies [4, 5, 12, 13]. In each preparation, only one experimental curve was determined.

Citalopram, GR55562, SB224289, U46619, 5-CT, α-methyl-5-HT (Tocris); serotonin, ketanserin, (–)-phenylephrine, acetylcholine (Sigma); LY393558 (gift from Eli Lilly). Acetylcholine, phenylephrine, citalopram, ketanserin, 5-CT, GR55562 and SB224289 were dissolved in distilled water and 5-HT in distilled water with a few drops of HCl. α-Methyl-5-HT was dissolved in water with DMSO, U46619 in ethanol and water. LY393558 was dissolved in DMSO that did not modify CRCs of agonists.

Results are given as mean ± SEM from *n* patients. Contractile responses were shown as percentages of the response to KCl 60 mM. GraphPad Prism 5.0 software was used to plot the mean data as sigmoidal CRCs. CRCs were used to determine potency (pEC_50_) and maximal effect (*E*_max_) of agonists. Antagonist potency (pA_2_) was determined according to [17]. pEC_50_ and pA_2_ values were not calculated when *E*_max_ values were < 50%.

Student's *t* test for unpaired data was used when one experimental group was compared to a control group. When ≥ 2 groups were compared to the same control, one-way analysis of variance (ANOVA) followed by Dunnett's test (post hoc test) was used. Post hoc tests were run only if *F* achieved the necessary level of statistical significance and there was no significant variance inhomogeneity. Differences were considered significant at *p* < 0.05.

## Results

Contractions (in mN) of 5-HT in endothelium-intact and endothelium-denuded hPAs were comparable among the groups (Table 1). Basal tension was not affected by 45-min pre-incubation with the antagonists, inhibitors and vehicles under study (not shown).

**Table 1:** Influence of antagonists and inhibitors on the constriction of human pulmonary arteries induced by serotonin (5-HT) receptor agonists

Group	*n*	Tension (mN)	pEC_50_	pA_2_	*E*_max_
5-HT	28	9.7 ± 1.0	6.20 ± 0.06		94 ± 4
+ Citalopram (1 μM)	11	8.9 ± 1.3	6.45 ± 0.08		87 ± 4
+ SB224289 (0.2 μM)	8	7.0 ± 1.9	5.71 ± 0.07***	7.02	90 ± 7
+ Citalopram (1 μM) + SB224289 (0.2 μM)	12	8.0 ± 1.3	5.58 ± 0.09***		66 ± 7***^,∆,a^
+ GR55562 (1 μM)	8	7.2 ± 2.0	5.48 ± 0.11***	6.63	76 ± 10
+ Citalopram (1 μM) + GR55562 (1 μM)	8	7.7 ± 1.2	5.30 ± 0.12***		51 ± 6***^,∆,a^
+ Ketanserin (0.1 μM)	4	7.7 ± 1.2	5.66 ± 0.19**	7.40	67 ± 12*
5-HT (-ENDO)	9	9.4 ± 1.2	5.77 ± 0.06***		92 ± 4
+ Citalopram (1 μM)-ENDO	5	7.6 ± 1.3	6.30 ± 0.15^$$,a^		90 ± 9
5-HT (DMSO)	9	8.5 ± 1.8	6.34 ± 0.08		82 ± 4
+ LY393558 (0.1 μM)	10	9.1 ± 1.7	5.00 ± 0.12^&&&,###,a^	8.25	50 ± 5^&&&,a^
5-CT (DMSO)	12	8.0 ± 1.0	6.36 ± 0.09		73 ± 6
+ SB224289 (0.2 μM)	4	10.0 ± 3.3	5.69 ± 0.27**^,a^	7.25	52 ± 13
+ GR55562 (1 μM)	6	10.9 ± 1.0	N.D		40 ± 10*
+ LY393558 (0.1 μM)	8	7.3 ± 2.0	N.D		34 ± 9**
α-Methyl-5-HT (DMSO)	7	10.5 ± 1.0	5.89 ± 0.08		79 ± 5
+ ketanserin (0.01 μM)	5	9.7 ± 2.3	N.D		48 ± 11*
+ LY393558 (0.1 μM)	7	10.5 ± 2.4	N.D		26 ± 6***

Data are expressed as mean ± SEM. Contractile responses are given in absolute terms and as percentages of the reference response to 60 mM KCl

*5-CT* 5-carboxamidotryptamine, *DMSO* dimethyl sulfoxide, -*ENDO* endothelium removal, *N.D.* could not be determined, *n* number of patients

*^,∆^*p* < 0.05; **^,$$^*p* < 0.01; ***^,&&&,###^*p* < 0.001, compared to the respective control (*5-HT, ^$^5-HT without endothelium, ^&^5-HT (DMSO), ^∆^5-HT + SB224289 or GR55562, ^#^citalopram + SB224289) as determined by one-way ANOVA followed by Dunnett’s post hoc test or ^a^Student’s *t* test (for full statistical results, i.e. *t*, *df*, and *p* values, see legends and results). Post hoc tests were run only if *F* achieved the necessary level of statistical significance and there was no significant variance inhomogeneity

**Fig. 1:**
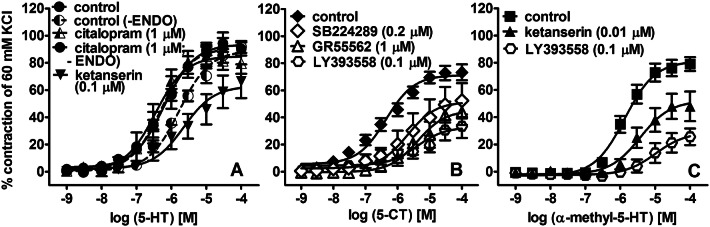
Influence of endothelium denudation (-ENDO) and/or citalopram (**a**), ketanserin (**a**, **c**), and SB224289, GR55562 (**b**) and LY393558 (**b**, **c**) on the vasoconstriction induced by serotonin (5-HT, A), 5-carboxamidotryptamine (5-CT, **b**) and α-methyl-5-HT (**c**) in isolated human pulmonary arteries. Results are expressed as percentages of the contraction induced by KCl and presented as mean ± SEM of 4–28 patients for each curve. **a** Statistical evaluation of the *E*_max_ values for control, control-ENDO, citalopram and ketanserin yielded *F*_(3,48)_ = 2.580 (*p* = 0.064) and *t* = 0.236; *df* = 12; *p* = 0.8175 for control-ENDO vs*.* citalopram-ENDO. The respective statistical tests for the pEC_50_ values yielded *F*_(3,48)_ = 12.86 (*p* < 0.0001; Dunnett’s test: *p* < 0.01 for ketanserin and for control-ENDO vs*.* control) and *t* = 3.909; *df* = 12; *p* = 0.0021. **b** Statistical evaluation of the *E*_max_ values for control, SB224289, GR55562 and LY393558 yielded *F*_(3,26)_ = 5.270 (*p* = 0.0057; Dunnett’s test: *p* < 0.05 for GR55562 and *p* < 0.01 for LY393558 vs*.* control). Comparison of the pEC_50_ values for control and SB224289 yielded *t* = 3.114; *df* = 14; *p* = 0.076. **c** Statistical evaluation of the *E*_max_ values for control, ketanserin and LY393558 yielded *F*_(2,16)_ = 15.91 (*p* = 0.0002; Dunnett’s test: *p* < 0.05 for ketanserin and *p* < 0.001 for LY393558 vs*.* control)

Serotonin and agonists of 5-HT_1B/1D_Rs and 5-HT_2A_Rs, 5-CT and α-methyl-5-HT, respectively, concentration-dependently contracted endothelium-intact hPAs (Fig. 1a–c). The rank order of potencies was 5-CT ≥ 5-HT > α-methyl-5-HT. The efficacy of 5-CT was lower than that of 5-HT and α-methyl-5-HT (*F*_(2,44)_ = 5.108; *p* = 0.01; Dunnett’s test: *p* < 0.01 for 5-HT vs. 5-CT; for pEC_50_ and *E*_max_ see Table 1).

As shown in Fig. 1a, endothelium removal inhibited the potency but not efficacy of the 5-HT-mediated contraction and caused a threefold rightward shift of its CRC. The SERT inhibitor citalopram (1 µM) did not influence the CRC for 5-HT in endothelium-intact hPAs, but enhanced the potency of 5-HT in endothelium-denuded preparations (for pEC_50_ values, see Table 1).

Blockade of 5-HT_2A_Rs by ketanserin (0.1 and 0.01 µM in Fig. 1a and c, respectively) shifted the CRC for 5-HT (by a factor of 3) and α-methyl-5-HT to the right and diminished *E*_max_ by 30% (for pEC_50_, *E*_max_ and pA_2_ values see Table 1).

**Fig. 2:**
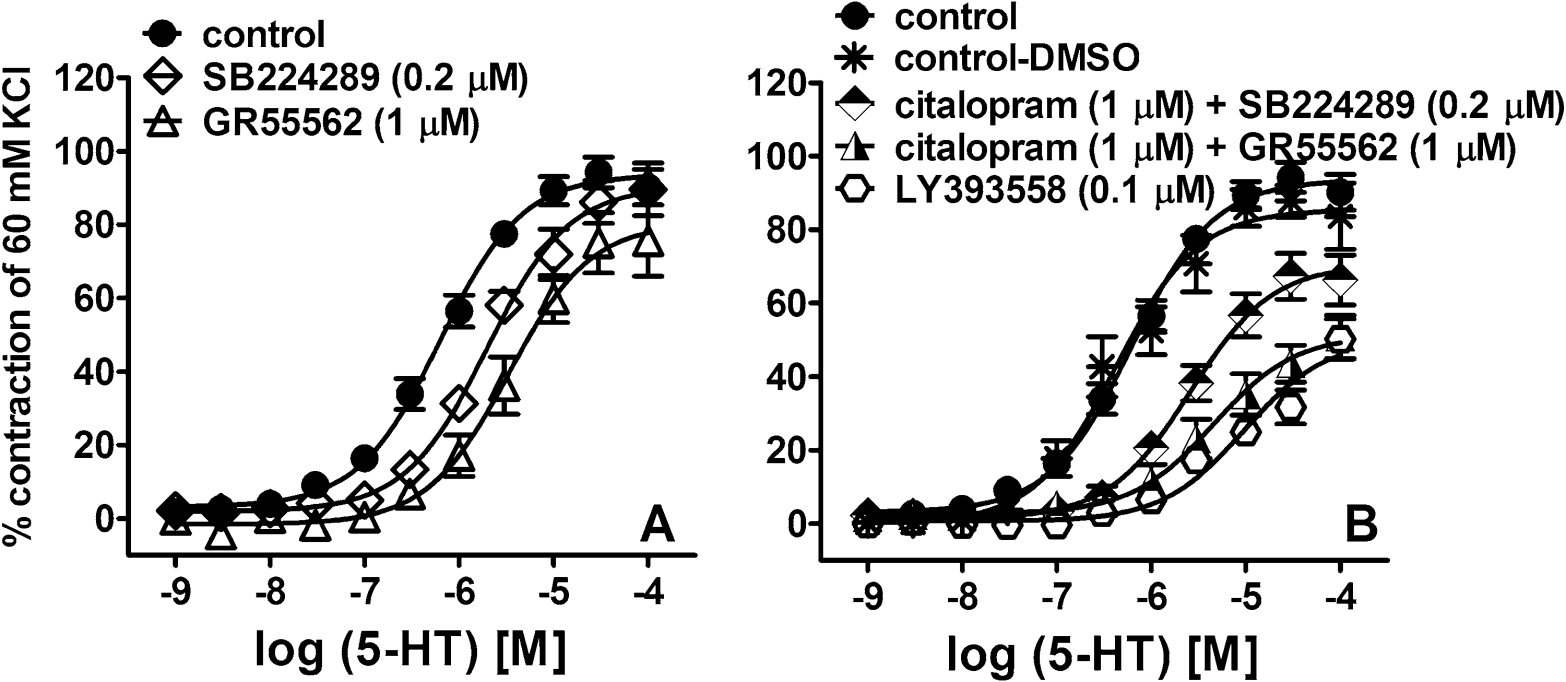
Influence of SB224289 and GR55562 (**a**), of combined administration of citalopram plus SB224289 or GR55562 (**b**) and of LY393558 (**b**) on the vasoconstriction induced by serotonin (5-HT) in isolated endothelium-intact human pulmonary arteries. Results are expressed as percentages of the contraction induced by KCl and presented as mean ± SEM of 8–28 patients for each curve. **a** Statistical evaluation of the *E*_max_ values for control, SB224289 and GR55562 yielded *F*_(2,41)_ = 2.022 ( *p* = 0.145); the corresponding statistical test for the pEC_50_ values yielded *F*_(2,41)_ = 21.98 (*p* < 0.0001; Dunnett’s test: *p* < 0.001 for both antagonists vs*.* control). **b** Statistical evaluation of control, citalopram + SB224289 and citalopram + GR55562 yielded *F*_(2,45)_ = 15.930 (*p* < 0.0001; Dunnett’s test: *p* < 0.001 for both antagonists vs*.* control) for *E*_max_ and *F*_(2,45)_ = 32.44 (*p* < 0.0001; Dunnett’s test: *p* < 0.001 for both antagonists *vs.* control) for pEC_50_. Statistical testing of control-DMSO vs*.* LY393558 yielded *t* = 4.594; *df* = 17; *p* = 0.0003 for *E*_max_ and *t* = 9.072; *df* = 17; *p* < 0.0001 for pEC_50_

With respect to a putative enhancement of 5-HT_1B_R antagonism by SERT blockade, four scenarios were considered, including administration of (i) 5-HT_1B_R antagonists (SB224289, GR55562) alone, (ii) citalopram alone, (iii) 5-HT_1B_R antagonists plus citalopram and (iv) LY393558 which combines 5-HT_1B_R antagonism and SERT blockade. SB224289 (0.2 µM) and GR55562 (1 µM) caused rightward shifts of the CRC for 5-HT by factors of 3 and 5, respectively (Fig. 2a) and reduced the efficacy of 5-CT by 30 and 45%, respectively (Fig. 1b). Citalopram (1 µM) had no effect when given alone (see above pEC_50_) but when co-administered with SB224289 (0.2 µM) or GR55562 (1 µM) reduced the potency and efficacy of the 5-HT-induced contraction (Fig. 2b). In the presence of citalopram, both antagonists only tended to diminish the potency of 5-HT (*t* = 1.042, *df* = 18; *p* = 0.31; *t* = 1.106, *df* = 14; *p* = 0.287, respectively) but markedly reduced its efficacy (*t* = 2.324, *df* = 18; *p* = 0.032; *t* = 2.144, *df* = 14; *p* = 0.050, respectively) when compared to the respective antagonists alone. LY393558 (0.1 µM) shifted to the right the CRCs for 5-HT (Fig. 2b), 5-CT (Fig. 1b) and α-methyl-5-HT (Fig. 1c) and reduced *E*_max_ values by 40, 55 and 70%, respectively. The antagonistic potency (pA_2_) for LY393558 against 5-HT was 8.3 and higher than that obtained with simultaneous administration of citalopram and SB224289 (*t* = 3.939, *df* = 20; *p* = 0.0008; for pEC_50_ and *E*_max_ values see Table 1).

**Fig. 3:**
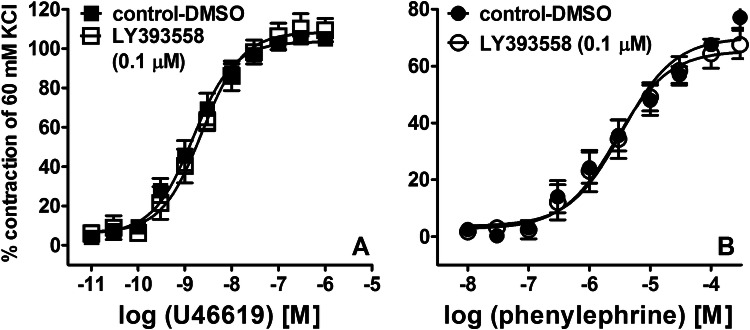
Influence of LY393558 on the vasoconstriction induced by U46619 (**a**) and phenylephrine (**b**) in isolated endothelium-intact human pulmonary arteries. Results are expressed as percentages of the contraction induced by KCl and presented as mean ± SEM of 5–6 patients for each curve. Statistical evaluation of the *E*_max_ values yielded *t* = 0.558; *df* = 9; *p* = 0.593 for **a** and *t* = 1.406; *df* = 10; *p* = 0.190 for **b**. The respective statistical tests for the pEC_50_ values yielded *t* = 1.571; *df* = 9; *p* = 0.150 and *t* = 0; *df* = 10; *p* = 1.0

LY393558 (0.1 µM) did not modify the vasoconstrictory effects on U46619 (pEC_50_ 8.80 ± 0.07, *n* = 5 vs. control: 8.60 ± 0.10, n = 6; *E*_max_ (relative to the KCl-induced response) 105 ± 3% vs. 109 ± 6%) and phenylephrine (pEC_50_ 5.50 ± 0.10, n = 6 vs. control: 5.50 ± 0.10, *n* = 6; *E*_max_ 77 ± 4% *vs*. 68 ± 5%) (Fig. 3).

## Discussion

The aim of the present study was to examine the effect of LY393558, a combined 5-HT_1B_R antagonist and SERT inhibitor, on the 5-HT receptor-mediated vasoconstriction of hPAs and to determine the underlying mechanism(s).

5-HT induced a concentration-dependent vasoconstriction of endothelium-intact hPAs. The results suggest that 5-HT_1B_Rs and 5-HT_2A_Rs are involved. First, 5-CT and α-methyl-5-HT (5-HT_1_ and 5-HT_2_ receptor agonists, respectively) mimicked the vasoconstriction of 5-HT with a rank order of potencies of 5-CT ≥ 5-HT > α-methyl-5-HT. 5-CT has been shown to be a more potent vasoconstrictor than 5-HT in main hPAs [4, 13] but they were equipotent in small hPAs [5]. Here we show that the efficacy of 5-CT was 20% lower than that of 5-HT, whilst other studies have shown the efficiency of 5-CT to be 50% less than that of 5-HT in hPAs [18]. In contrast to hPAs, responses to 5-HT were less in rat [12] and more potent in mouse PAs [13]. Second, the 5-HT_1B_R antagonists SB224289 (has no affinity for the SERT; [12]) and GR55562 (5-HT_1B/1D_-selective antagonist; [5]) inhibited the 5-HT-induced vasoconstriction with pA_2_ values of 7.0 and 6.6, respectively, that are consistent with their respective values in large hPAs [4] and slightly lower than those in small hPAs [5]. Moreover, their inhibitory potencies were more potent against the contraction induced by the 5-HT_1_R agonist than that induced by 5-HT. Similarly, the 5-HT_2A_R antagonist ketanserin reduced the response to 5-HT with a pA_2_ of 7.4 but had a much stronger effect against the 5-HT_2_R agonist. pA_2_ values could not be calculated in any case, as described before due to the biphasic nature of the 5-HT responses in the presence of ketanserin [4, 5].

SERT inhibitors can cause extracellular accumulation of 5-HT and subsequent 5-HTR activation and therefore potentiate the effects of 5-HT [12, 19]. Indeed the potency of 5-HT is reduced in the PAs from SERT^+^ mice which over-express SERT compared to wild type mice [13] and in normoxic SD and FH vessels, and this is associated with an enhanced expression of SERT in comparison to their hypoxic counterparts [12]. Hence we determined the influence of SERT inhibition on the 5-HT-induced vasoconstriction. We used citalopram 1 µM as SERT inhibitor (because it does not affect 5-HT receptors directly [19]), which, however, did not change the effect of 5-HT. Our results differ from those in intact rat intralobar PAs in which citalopram 0.1 µM potentiated the 5-HT-induced vasoconstriction [19]. Since this effect did not occur in their endothelium-denuded counterparts [19], we performed additional experiments in endothelium-denuded rings and under these circumstances citalopram shifted the CRC for 5-HT to the left. The reason why removal of endothelium attenuated the effect of 5-HT in our hands is unclear. One should keep in mind that potentiation, inhibition or lack of effect of citalopram on 5-HT-induced constriction depends on its concentration, vascular bed (pulmonary vs systemic), vessel size, intactness of endothelium and oxygen supply (normoxia vs hypoxia) [12, 13, 19].

In experiments on endothelium-intact hPAs we observed a synergic interaction between SERT inhibition and 5-HT_1B_R antagonism. Thus, citalopram at a concentration that failed to modify the 5-HT effect in hPAs more strongly inhibited the 5-HT-induced vasoconstriction when given together with one of the 5-HT_1B_R antagonists SB224289 or GR55562 compared to SB224289 or GR55562 alone. Our data conform to previous experiments on PAs from SD rats, in which SB224289 exhibited an interaction with an inactive concentration of the SERT inhibitor fluoxetine against the 5-HT-induced contraction [12]. The mechanism of this interaction is so far not fully understood and may have something to do with the fact that 5-HT_1B_Rs and SERT appear to cooperate in the contractile (and proliferative) effects of 5-HT in the pulmonary circulation [2].

LY393558 combines 5-HT_1B_R antagonism and SERT inhibition [12, 13, 16] with *K*_i_ values of ~ 1 nM in each case [15, 16]. We are the first to show that LY393558 strongly reduced both potency and efficacy of 5-HT-induced vasoconstriction in hPAs. Its antagonistic potency was similar to that of GR55562 plus citalopram and even stronger than that of SB224289 plus citalopram. Targeting both SERT and 5-HT_1B_Rs is suggested to be a novel therapeutic approach to PAH [2, 12]. So far, it has been shown that LY393558 strongly reduced the potency and maximal effect of the 5-HT-induced constriction of PAs from normoxic and hypoxic SD and FH rats (its pA_2_ value was not determined because *E*_max_ values were below 50%); its chronic administration was more effective in the prevention and reversal of PAH in two mouse PAH models than that of citalopram [12, 13]. Interestingly, LY393558 reduced the 5-HT-induced proliferation of hPASMCs from idiopathic PAH patients stimulated by 5-HT [13].

What is the mechanism behind the effect of LY393558 on the contractile response in hPAs? LY393558 reduced the vasoconstriction induced by the 5-HT_1_R agonist 5-CT to a similar extent like GR55562 1 µM and even more strongly than SB224289 0.2 µM, confirming its antagonistic activity against 5-HT_1B_Rs. Moreover, LY393558 is an antagonist at 5-HT_2A_Rs (pK_i_ ~ 100 nM; [16]) and this property appears to contribute to the overall effect of LY393558 against the 5-HT-mediated constriction. Thus, the vasoconstriction of hPAs induced by the 5-HT_2_R agonist α-methyl-5-HT was diminished by LY393558 by 50% and by the standard 5-HT_2A_R antagonist ketanserin 0.01 µM by 30%.

Although the selectivity of LY393558 for some 5-HT-R subtypes has been described [16], to the best of our knowledge no data to its specificity have been published. Our data show that the drug does not possess a marked affinity at human α_1_ and TP receptors (pA_2_ < 7.0).

Finally, we would like to underline two limitations of our study. (1) One may criticize the lack of selectivity of 5-HT-R agonists and antagonists. However, according to the IUPHAR/BPS Guide to PHARMACOLOGY the 5-HT_2A_R agonist α-methyl-5-HT does not possess any affinity at 5-HT_1B_Rs and GR55562 is a selective antagonist of 5-HT_1B_Rs [20]. Thus, we used at least one selective agonist or antagonist of 5-HT_2A_Rs and 5-HT_1B_Rs that allowed us to conclude that both 5-HT-R subtypes are involved in the effect of LY393558. (2) SERT has been suggested to play a role in the development of PAH in an estrogen-dependent manner both in experimental models and in humans [2]. Our experiments were performed in tissues obtained from patients (mainly males) without PAH. We cannot exclude that the role of citalopram would be more marked in pulmonary arteries isolated from women suffering from PAH.

In summary, LY393558 reduced the 5-HT-induced contraction antagonizing 5-HT_1B_Rs and 5-HT_2A_Rs and probably due to synergic interaction between SERT inhibition and 5-HT_1B_R antagonism. Since LY393558 (given acutely and/or chronically) has been shown previously to inhibit the 5-HT-induced hPASMCs proliferation and to prevent and to reverse experimental PAH it might represent a future option in the PAH therapy.
